# 744. Infectious Diseases Providers Perspectives Around the Use of Outpatient Parenteral Antibiotic Therapy (OPAT) for Persons Who Inject Drugs (PWID), US, 2022

**DOI:** 10.1093/ofid/ofac492.035

**Published:** 2022-12-15

**Authors:** Daniel A Solomon, Alison M Beieler, Sera Levy, Ellen Eaton, Monica K Sikka, Alice C Thornton, Shireesha Dhanireddy

**Affiliations:** Brigham and Women's Hospital / Harvard Medical School, Boston, MA; University of Washington, Seattle, Washington; University of Alabama at Birmingham, Birmingham, Alabama; University of Alabama, Birmingham, Birmingham, Alabama; Oregon Health and Science University, Portland, Oregon; University of Kentucky, Lexington, Kentucky; University of Washington, Seattle, Washington

## Abstract

**Background:**

Injection drug use-related infections are life threatening and may require prolonged courses of antibiotic therapy. There is no consensus on best practices for serious infections when patients are stable for discharge. Outpatient parenteral antimicrobial therapy (OPAT) has been shown to be feasible for people who inject drugs (PWID) especially when paired with medication for opioid use disorder. We set out to determine the current practice patterns and attitudes of infectious diseases (ID) clinicians in the United States regarding the use of OPAT for PWID.

**Methods:**

We surveyed ID clinicians between January 2022 and March 2022. Topics focused on PWID including OPAT access, barriers to inclusion in OPAT, and factors clinicians considered for OPAT success. Responses from those who perceived PWID were eligible for OPAT (PWID-E) were compared to those who believed PWID were ineligible (PWID-I) using the Pearson chi-squared test.

**Results:**

Of 239 clinician respondents, 80% were MD, 6% DO, 5% NP, 2% PA, and 7% Pharmacists. 187 (78.2%) work at an academic practice and 179 (75%) work in an urban setting. 182 (76%) respondents have access to inpatient addiction medicine services and 206 (86%) reported availability of outpatient addiction treatment, but only 86 (36%) described patient access to outpatient services as “good” or “excellent.”

Respondents were categorized as PWID-E (n=171, 72%) and PWID-I (n=68, 28%). Of the PWID-E group, only 48 (28%) have an institutional policy for determining eligibility. There was no significant difference in practice setting or access to addiction services between PWID-E and PWID-I. Access to inpatient and outpatient social work/case management was significantly higher for PWID-E. (Table 1).

Clinicians reporting PWID-I were more likely to cite risk of tampering with PICC (76.5% vs 62.6%, p = 0.04) and medical-legal risk (47.1% vs 19.3%, p < 0.001) as barriers to OPAT. (Table 2).

**Conclusion:**

A high proportion of respondents to this national survey offer OPAT for PWID, but only a minority have an institutional policy for eligibility. Guidelines that outline a framework for discharge decision making, education regarding medical and legal risk, and development of best practices may help to standardize care across settings.

**Disclosures:**

**Ellen Eaton, MD, MPH**, Gilead HIV Research Scholar: Grant/Research Support|Gilead HIV research scholar: Grant/Research Support **Monica K. Sikka, MD**, F2G: Site research investigator.

